# Risks Associated with Surgical Treatment for Appendicitis in Hematologic Patients

**DOI:** 10.3390/cancers15205049

**Published:** 2023-10-19

**Authors:** Seung Hyun Lee, Sung-Soo Park, Ho Seok Seo

**Affiliations:** 1 Department of Surgery, College of Medicine, The Catholic University of Korea, Seoul 06591, Republic of Korea; lshyun226@nate.com; 2 Division of Hematology, Department of Internal Medicine, Seoul St. Mary’s Hospital, College of Medicine, The Catholic University of Korea, Seoul 06591, Republic of Korea; sspark@catholic.ac.kr; 3 Division of Gastrointestinal Surgery, Department of Surgery, Seoul St. Mary’s Hospital, College of Medicine, The Catholic University of Korea, Seoul 06591, Republic of Korea

**Keywords:** appendicitis, appendectomy, hematologic disease

## Abstract

**Simple Summary:**

Appendicitis is a common condition necessitating emergency surgery. Individuals with hematologic disorders often have abnormalities in blood counts, which can act as risk factors for surgical complications. This study explores surgical risks in appendicitis patients with hematologic disorders, using a retrospective analysis from January 2000 to June 2021. Among the 131 identified patients, 89 were studied; 75 underwent surgery, while 14 received non-surgical treatments. No significant relationship was found between clinical characteristics, hematologic disease risk, severity of appendicitis, and surgical complications. Improved preoperative absolute neutrophil count and platelet counts were higher than those of initial findings in patients without surgical complications. Lower preoperative absolute neutrophil counts and platelet counts were correlated with extended hospital stays. The findings suggest that for hematologic patients with appendicitis, meticulous preoperative laboratory evaluations followed by minimally invasive appendectomy could be a safer approach without increasing the risk of severe complications compared to non-surgical management.

**Abstract:**

Appendicitis is a prevalent surgical emergency. Although appendectomy has traditionally been the go-to treatment, recent studies suggest antibiotics can be equally effective for uncomplicated cases. However, evidence is scant regarding patients with hematologic disorders. This study delves into the surgical risks tied to appendicitis in patients with underlying hematologic conditions. A retrospective analysis was carried out on patients diagnosed with appendicitis and hematologic disorders from January 2000 to June 2021. Patients were pinpointed using ICD-10 diagnostic codes, and surgical procedures were identified based on the hospital’s surgical fee codes. Hematologic conditions were sorted into risk levels, and patient treatments were scrutinized. Among the 131 initially identified patients, 89 were included in the study. Out of these, 75 underwent surgical procedures, while 14 received non-surgical treatments. The surgical group displayed better preoperative laboratory outcomes. Clinical characteristics, hematologic disease risk, and severity of appendicitis appeared not to be related to surgical complications. Patients without surgical complications showed improvement in preoperative absolute neutrophil count (ANC) and platelet counts. Lower preoperative ANCs and platelet counts were associated with extended hospital stays. For patients with hematologic disorders diagnosed with appendicitis, thorough preoperative laboratory evaluations followed by minimally invasive appendectomy appear to be a safe route without heightening the risk of severe complications compared to non-surgical management.

## 1. Introduction

Appendicitis, characterized by inflammation of the appendix, remains one of the most common surgical emergencies. In the Republic of Korea, the annual diagnosis rate stands at approximately 227.1 per 100,000 individuals, translating to a lifetime incidence of around 16.3% [1]. Various therapeutic approaches are available for treating appendicitis, including surgical interventions like appendectomy, conservative management with antibiotics, and percutaneous drainage in the presence of an appendiceal abscess. Currently, appendectomy is considered the gold standard, being performed in 60–80% of cases [1,2,3]. It has been observed that surgical treatment for appendicitis results in fewer re-operations compared to conservative management [4,5]. However, recent research suggests that antibiotics for uncomplicated appendicitis can be just as effective as an appendectomy [6,7]. In countries with well-established surgical systems, the inclination is typically toward surgical intervention [8].

Among those diagnosed with appendicitis, a subset have concomitant hematologic disorders. Such patients often exhibit atypical complete blood count (CBC) findings [9]. While it might be postulated that these irregularities heighten surgical risks, including complications and mortality, some studies indicate that the differential risk may be less pronounced than initially believed [10]. In addition, ratios derived from CBC parameters (such as the systemic immune inflammatory index) and other biomarkers such as ischemia-modified albumin have been reported as potential diagnostic tools, particularly in pediatric appendicitis, where diagnosis can be challenging [11,12,13,14].

In this study, we aim to evaluate the risks associated with surgical treatment in patients with appendicitis who also have underlying hematologic disorders and analyze the prognosis based on preoperative blood test results.

## 2. Materials and Methods

### 2.1. Study Design and Cohort

We conducted a retrospective analysis of patients diagnosed with appendicitis who also had concomitant hematologic conditions at Seoul St. Mary’s Hospital from January 2000 to June 2021. Both conditions were identified using ICD-10 diagnostic codes. Hematologic diseases were defined using codes C81-96 (malignant neoplasms of lymphoid, hematopoietic, and related tissue), D693 (idiopathic thrombocytopenic purpura), D61 (other aplastic anemias), D46 (myelodysplastic syndromes), and D47 (neoplasms of uncertain or unknown behavior of lymphoid, hematopoietic, and related tissue). Appendicitis was identified using codes K35–37. Surgical procedures were determined based on the surgical fee codes at Seoul St. Mary’s Hospital: JP2861–2863 for appendectomy, and JP267101 and JP2673 for right hemicolectomy (RHC) and ileocecectomy, respectively.

### 2.2. Hematologic Disease Classification

Hematologic conditions were categorized as low, intermediate, high-risk, or other, as per the referenced classification [15]. This classification primarily caters to patients post-hematopoietic stem cell transplantation, either following myeloablative conditioning (MAC) or reduced intensity conditioning (RIC). An initial model for overall survival was constructed, incorporating factors such as age, donor and recipient gender, human leukocyte antigen compatibility, and disease status. Patients were then segregated based on MAC and RIC, further stratified by disease status and hazard ratio. A detailed breakdown of this classification is presented in Supplementary Table S1, showcasing the distribution of hematologic disease patients in our cohort by risk level.

### 2.3. Patient Selection and Treatment

**Figure 1:**
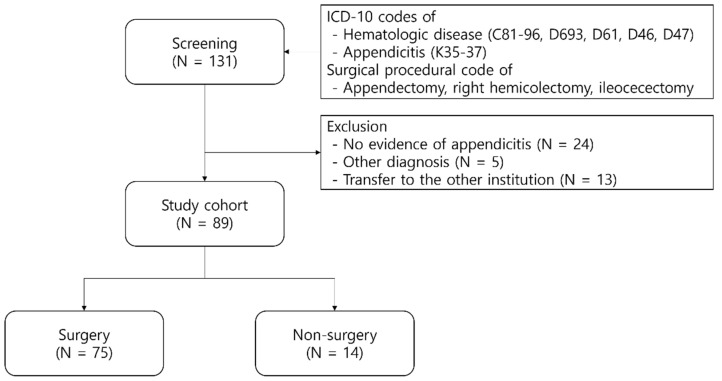
Flow sheet of patient inclusion.

From the diagnostic codes, 131 patients were initially identified. Of these, 24 were verified to lack evidence of appendicitis. Five patients, diagnosed with other conditions such as diverticulitis and colitis, were excluded. An additional 13 were transferred to different institutions, leading to a loss of follow-up. Of the 89 patients retained in the study, 75 underwent surgical procedures, while 14 were managed non-surgically. Non-surgical management is composed of intravenous antibiotic regimens and interventions like percutaneous abscess drainage. Surgical interventions included appendectomy, ileocecectomy, and RHC, with both laparoscopic and open techniques employed (Figure 1).

### 2.4. Terminologies Used in the Study

“Initial laboratory findings” refer to the first set of tests administered upon a patient’s presentation to the hospital following the onset of appendicitis symptoms. This includes evaluations of absolute neutrophil count (ANC), hemoglobin (Hb), and platelet (PLT) counts. “Preoperative laboratory findings” denote the latest tests performed before surgery, encompassing ANC, Hb, and PLT counts. This terminology is relevant for patients assessed multiple times post-admission. “In-hospital mortality” is characterized as any patient death occurring post-appendicitis diagnosis but prior to hospital discharge.

### 2.5. Statistical Analysis

For non-parametric continuous variables, the Mann-Whitney U test was used. The data were summarized with the median [interquartile range]. Categorical data were analyzed using the chi-square test or Fisher’s exact test. Multivariate analyses were performed using logistic regressions for reporting odds ratios (ORs). Differences with a two-sided *p* value < 0.05 were considered to be statistically significant. SPSS (ver.24; SPSS, Inc., Chicago, IL, USA) for Windows software was used for statistical analysis.

### 2.6. Ethical Approval and Consent to Participate

The institutional review board of the College of Medicine at the Catholic University of Korea approved this study (approval no. KC23RISI0693). We anonymized and de-identified all patient records before analysis.

## 3. Results

Table 1 presents a comparison of clinical characteristics between patients who underwent surgery and those who did not. The majority in both groups were diagnosed with acute myeloid leukemia and were predominantly categorized as intermediate risk. This trend is consistent with the general population of patients with hematologic diseases and appears unrelated to appendicitis [15]. No significant differences were observed in age, Eastern Cooperative Oncology Group (ECOG) performance status, or concurrent diseases between the two groups.

Table 2 displays the perioperative parameters, including appendicitis-related information and blood test results at the time of appendicitis diagnosis. The surgery group had a significantly higher initial ANC than the non-surgery group (*p* = 0.001), while the initial Hb level and PLT counts showed no statistical difference between the groups (*p* = 0.261 and 0.127, respectively). Interestingly, the total hospital stay was significantly shorter in the surgery group (*p* = 0.026), and the in-hospital mortality rate was relatively lower in the surgery group, although not statistically significant (14.3% vs. 2.7%, *p* = 0.115).

To analyze the impact of hematologic disease risk on surgical outcomes, clinical characteristics and short-term surgical outcomes were examined based on hematologic disease risk in the patients who underwent surgery. Most parameters, such as age, ECOG performance status, and severity of appendicitis, showed no statistical differences across different risk categories. Also, there was no difference in terms of initial or preoperative ANC, Hb, or PLT count between the groups. The complication rates within each risk group are as follows: low risk at 9.7%, intermediate risk at 11.4%, and high risk at 28.6%, without statistical difference (*p* = 0.380) (Supplementary Table S2).

Clinical characteristics including age, sex, BMI, ECOG score, and even the type of hematologic disease seemed not to be related to surgical complications (Supplementary Table S3). Similarly, hematologic disease risk, type of appendicitis, type of surgery, surgical approach, and even the preoperative ANC and PLT count seemed not to be related to surgical complications. Notably, although surgical complications would prolong the postoperative hospital stay, they were not related to in-hospital mortality (Table 3). Supplemental Table S4 provides details on the complications observed in patients, which were classified according to the Clavien-Dindo system [16]. Among the ten patients who experienced surgical complications, issues like wound infections, fluid collections, postoperative ileus, pneumonia, and fever were encountered. All these complications were either grade 1 or 2, with no instances of more severe surgical complications.

In terms of preoperative laboratory adjustments, patients without complications showed an improvement in preoperative ANC and PLT levels compared to initial levels (Table 3). In contrast, patients with complications did not show any improvement in those findings. These results demonstrate the potential association between proper preoperative adjustment of ANC and PLT levels and a reduction in surgical complications.

Perioperative parameters were compared according to the preoperative laboratory findings, specifically focusing on ANC and PLT values in the patients who underwent surgery. These were categorized based on thresholds of 1.00 (10^9^/L) for ANC and 100 (10^9^/L) for PLTs. Patients with ANC values below 1.00 had a longer total and postoperative hospital stay than those above 1.00 (*p* = 0.007 and *p* = 0.006, respectively). Similarly, patients with PLT counts below 100 had a more extended total and postoperative hospital stay compared to those with higher counts (*p* < 0.001 and *p* < 0.001, respectively). An interesting observation was the in-hospital mortality rate, which increased as PLT counts decreased. The mortality rate was 9.5% for those with PLT counts below 100, while it was 0% for those with higher counts (*p* = 0.022) (Table 4).

Lastly, Table 5 focuses on the analysis of risk factors that might extend hospital stays beyond 7 days postoperation in the patients who underwent surgery. Through both univariate and multivariate analyses, factors such as the risk associated with hematologic diseases, the surgical method used, and ANC and PLT counts were identified as independent risk factors for prolonged hospital stays.

## 4. Discussion

Patients with hematologic disorders often exhibit abnormal CBCs. Historically, surgical interventions in these patients with unusual CBC results have been linked to increased postoperative complications. For instance, thrombocytopenia frequently requires transfusions and is associated with higher 30-day mortality and a range of complications, from pulmonary to thromboembolic events [17]. Neutropenia has been connected to increased postoperative mortality and a rise in major morbidities, especially infections [18]. Similarly, anemia elevates 30-day mortality, often necessitating transfusion interventions [19,20]. Hematologic disorders inherently raise surgical risks, including increased mortality, multi-organ complications, and reduced long-term survival [21,22].

Several studies have explored the relationship between laboratory findings and appendicitis, with a significant amount of research focused on pediatric appendicitis, where diagnosis is more challenging compared to adults. The efficacy of computed tomography in diagnosing pediatric appendicitis is relatively lower; hence, ultrasound is commonly utilized [23]. To improve diagnostic accuracy, leveraging laboratory findings, particularly the systemic immune inflammation index, has been found to be beneficial [11,23]. Moreover, the role of ischemia-modified albumin has been reported to assist in diagnosing pediatric appendicitis [12]. The utility of such biomarkers extends beyond the diagnostic realm. A study highlighted the usefulness of interleukin-6 as a biomarker related to patient prognosis, such as hospital stay duration in pediatric appendicitis [13,14]. These laboratory findings are valuable in diagnosing and prognosticating in pediatric patients with atypical symptoms or situations and are believed to be similarly useful in patients with hematologic diseases, who also present atypically. Indeed, in this study, a decrease in preoperative ANC and PLT counts was associated with an extended postoperative hospital stay duration.

With the advent of laparoscopy, minimally invasive surgery has emerged, leading to fewer surgical complications and enhanced quality of life [24]. Such minimally invasive approaches have become a viable option for surgical intervention even in high-risk patients [25]. Appendectomy, as a representative minimally invasive procedure, is deemed particularly beneficial for patients with concurrent hematologic diseases, minimizing surgical risks while maximizing the benefits [26]. Several studies suggest that the correlation between abnormal CBC and complications in appendectomies might be weak [10,27,28,29,30,31]. In this study, most surgeries were performed using minimally invasive techniques, and there were no significant complications associated with the surgery.

Our study aimed to explore the surgical risks for patients with hematologic disorders diagnosed with appendicitis. Our findings, outlined in Table 2 and Table 3, revealed that patients who underwent surgical interventions had better preoperative laboratory findings and experienced shorter hospital stays compared to patients who received non-surgical treatment. The occurrence of postoperative complications did not show a significant association with hematologic disease risk or preoperative laboratory findings. Moreover, even when postoperative complications occurred, they were limited in number and, according to the Clavien-Dindo classification, were confined to grades 1–2, with only the postoperative hospital stay being prolonged and no difference in in-hospital mortality. The two mortalities in the surgery cohort, as presented in Table 2, were attributed to the exacerbation of their primary hematologic condition following a successful appendectomy rather than the surgical intervention itself. These results suggest that aggressive surgical intervention could be beneficial for patient outcomes when appendicitis is diagnosed in patients with hematologic diseases.

In terms of preoperative laboratory findings, there was an improvement in preoperative ANC, Hb, and PLT levels compared to the initial findings in patients with no complications. This result suggests the imperative for meticulous preoperative evaluations and potential interventions, such as transfusions, to mitigate complications. In summary, appropriate CBC adjustments coupled with prompt diagnosis can be implemented to aid in reducing complications through minimally invasive surgery.

This study, while insightful, is not without limitations. Its single-institution nature and relatively modest sample size of 89 patients may limit the generalizability of the findings. The lack of follow-up for 13 patients transferred to other institutions, along with the inherent variability in patient responses based on the progression and treatment status of their hematologic conditions, further compound these limitations. Additionally, the evolving treatments over the two-decade span of this study introduce another layer of variability. The lack of a standardized criterion for surgery versus conservative care for appendicitis introduces potential biases in treatment outcomes. Moreover, the difference in patient numbers between the groups might cause biases. Due to the high heterogeneity of the cohort, certain information, such as antibiotic usage, the patient’s general condition, the severity of appendicitis, or the character of appendicitis in consideration of other adjacent organs, could not be analyzed individually. Nevertheless, the novelty of the study lies in its pioneering exploration of surgical risks for appendicitis in patients with hematologic disorders. This study could serve as a foundation for the development of tailored guidelines for this unique patient cohort.

## 5. Conclusions

For patients diagnosed with appendicitis who also have underlying hematologic conditions, careful preoperative laboratory adjustments and choosing a minimally invasive appendectomy seem to be safe options. These approaches do not increase the incidence of severe complications when compared to conservative treatments.

## Figures and Tables

**Table 1:** Clinical characteristics according to the type of treatment.

Variables	Non-Surgery (*n* = 14)	Surgery (*n* = 75)	*p*-Value
Age (years)	39.5 [10.5–62.25]	44.0 [29–60]	0.370
Sex			0.561
Male	7 (50)	45 (60)	
Female	7 (50)	30 (40)	
BMI (kg/m^2^)	18.8 [17.1–23.3]	22.0 [20.2–23.9]	0.047
ECOG score			0.261
0 or 1	11 (78.6)	67 (89.3)	
2 or higher	3 (21.4)	8 (10.7)	
Comorbidities			
HBP	2 (14.3)	9 (12)	0.811
DM	0 (0)	7 (9.3)	0.234
Cardiovascular	1 (7.1)	4 (5.3)	0.787
Pulmonary	1 (7.1)	4 (5.3)	0.787
Hepatic	0 (0)	7 (9.3)	0.234
Renal	0 (0)	5 (6.7)	0.320
Cerebral	0 (0)	1 (1.3)	0.664
Other malignancy	0 (0)	1 (1.3)	0.664
Others	1 (7.1)	10 (13.3)	0.518
Hematologic disease			0.708
AML	8 (57.1)	28 (37.3)	
ALL	3 (21.4)	12 (16)	
MDS	0 (0)	2 (2.7)	
Lymphoma	2 (14.3)	9 (12)	
MM	0 (0)	7 (9.3)	
CML	1 (7.1)	13 (17.3)	
CLL	0 (0)	2 (2.7)	
ITP	0 (0)	2 (2.7)	
Hematologic disease risk			0.850
Low	5 (35.7)	31 (41.3)	
Intermediate	8 (57.1)	35 (46.7)	
High	1 (7.1)	7 (9.3)	
Others	0 (0)	2 (2.7)	

Data are given as numbers (%) and medians [interquartile range]. BMI, body mass index; ECOG, Eastern Cooperative Oncology Group; HBP, hypertension; DM, diabetes mellitus; AML, acute myeloid leukemia; ALL, acute lymphoblastic leukemia; MDS, myelodysplastic syndrome; MM, multiple myeloma; CML, chronic myeloid leukemia; CLL, chronic lymphoblastic leukemia; ITP, immune thrombocytopenic purpura.

**Table 2:** Perioperative parameters according to the type of treatment.

Variables	Non-Surgery (*n* = 14)	Surgery (*n* = 75)	*p*-Value
Diagnosis area			0.492
Emergency room	10 (71.4)	59 (78.7)	
Inpatient (hematology)	4 (28.6)	13 (17.3)	
Outpatient clinic	0 (0)	3 (4)	
Type of appendicitis			0.355
Simple	7 (50)	49 (65.3)	
Perforated	2 (14.3)	12 (16)	
Periappendiceal abscess	5 (35.7)	14 (18.7)	
Appendicolith	1 (8.3)	5 (6.7)	0.832
Initial ANC (10^9^/L)			0.001
>1.00	6 (42.9)	61 (81.3)	
0.50~1.00	1 (7.1)	6 (8)	
<0.50	7 (50)	8 (10.7)	
Initial Hb (g/dL)			0.261
<8.0	3 (21.4)	8 (10.7)	
Initial PLT count (10^9^/L)			0.127
>100	6 (42.9)	53 (70.7)	
50~100	3 (21.4)	9 (12)	
<50	5 (35.7)	13 (17.3)	
Total hospital stay (days)	16 [6–30.5]	5 [3–19]	0.026
In-hospital mortality	2 (14.3)	2 (2.7)	0.115

Data are given as numbers (%) and medians [interquartile range]. ANC, absolute neutrophil count; Hb, hemoglobin; PLT, platelet.

**Table 3:** Perioperative parameters related to the surgical complications.

Variables	No Complication (*n* = 65)	Complication (*n* = 10)	*p*-Value
Hematologic disease risk			0.240
Low	28 (43.1)	3 (30)	
Intermediate	31 (47.7)	4 (40)	
High	5 (7.7)	2 (20)	
Others	1 (1.5)	1 (10)	
Type of appendicitis			0.266
Simple	41 (63.1)	8 (80)	
Perforated	10 (15.4)	2 (20)	
Periappendiceal abscess	14 (21.5)	0 (0)	
Appendicolith	3 (4.6)	2 (20)	
Type of surgery			0.455
Appendectomy	56 (86.2)	10 (100)	
Ileocecectomy	7 (10.8)	0 (0)	
Right hemicolectomy	2 (3.1)	0 (0)	
Approach			0.481
Open	3 (4.6)	1 (10)	
Laparoscopy	62 (95.4)	9 (90)	
Drain	34 (52.3)	4 (40)	0.469
OP time (min)	57 [45.0–87.5]	75 [48.5–87.5]	0.569
EBL (mL)	20 [10–50]	10 [5–36.5]	0.190
Initial ANC (10^9^/L)			0.968
>1.00	53 (81.5)	8 (80)	
0.50~1.00	5 (7.7)	1 (10)	
<0.50	7 (10.8)	1 (10)	
Initial Hb (g/dL)			0.240
<8.0	8 (12.3)	0 (0)	
Initial PLT count (10^9^/L)			0.455
>100	45 (69.2)	8 (80)	
50~100	9 (13.8)	0 (0)	
<50	11 (16.9)	2 (20)	
Preoperative ANC (10^9^/L)			0.407
>1.00	58 (89.2)	8 (80)	
0.50~1.00	2 (3.1)	0 (0)	
<0.50	5 (7.7)	2 (20)	
Preoperative Hb (g/dL)			0.574
<8.0	2 (3.1)	0 (0)	
Preoperative PLT count (10^9^/L)			0.968
>100	47 (72.3)	7 (70)	
50~100	13 (20)	2 (20)	
<50	5 (7.7)	1 (10)	
Total hospital stay (days)	5 [3–19.5]	8.5 [6.5–17.0]	0.210
Postoperative hospital stay (days)	4 [2.5–7.0]	7 [4–9.0]	0.067
In-hospital mortality	2 (3.1)	0 (0)	0.574

Data are given as numbers (%) and medians [interquartile range]. OP, operation; EBL, estimated blood loss; ANC, absolute neutrophil count; Hb, hemoglobin; PLT, platelet.

**Table 4:** Comparison of perioperative parameters according to the preoperative laboratory findings.

Variables	ANC ≥ 1.00 (10^9^/L) (*n* = 66)	ANC < 1.00 (10^9^/L) (*n* = 9)	*p*-Value	PLTs ≥ 100 (10^9^/L) (*n* = 54)	PLTs < 100 (10^9^/L) (*n* = 21)	*p*-Value
Type of surgery			0.000			0.070
Appendectomy	59 (89.4)	7 (77.8)		49 (90.7)	17 (81)	
Ileocecectomy	7 (10.6)	0 (0)		5 (9.3)	2 (9.5)	
Right hemicolectomy	0 (0)	2 (22.2)		0 (0)	2 (9.5)	
Approach			0.411			0.314
Open	3 (4.5)	1 (11.1)		2 (3.7)	2 (9.5)	
Laparoscopy	63 (95.5)	8 (88.9)		52 (96.3)	19 (90.5)	
Drain	33 (50)	5 (55.6)	0.597	23 (42.6)	15 (71.4)	0.482
OP time (min)	64.5 [45.0–91.5]	55.0 [45.0–85.0]	0.961	64.5 [45.0–81.5]	60.0 [50.0–102.5]	0.285
EBL (mL)	20.0 [10.0–50.0]	10.0 [5.0–65.0]	0.530	20.0 [8.5–50.0]	20.0 [5.0–60.0]	0.406
Total hospital stay (days)	4.5 [3.0–13.5]	28.0 [10.0–49.5]	0.007	4.0 [3.0–8.0]	20.0 [7.5–40.0]	<0.001
Postoperative hospital stay (days)	4 [2.5–7.0]	10.0 [4.5–44.5]	0.006	3.5 [2.0–6.0]	7.0 [5.0–13.0]	<0.001
Complication	8 (12.1)	2 (22.2)	0.403	7 (13)	3 (14.3)	0.880
In-hospital mortality	1 (1.5)	1 (11.1)	0.094	0 (0)	2 (9.5)	0.022

Data are given as numbers (%) and medians [interquartile range]. ANC, absolute neutrophil count; PLT, platelet; OP, operation; EBL, estimated blood loss.

**Table 5:** Risk factors for prolonged postoperative hospital stay (over 7 days postoperation).

	Univariate				Multivariate			
Variables	OR	LCI	HCI	*p*-Value	OR	LCI	HCI	*p*-Value
Age	0.998	0.954	1.043	0.929				
Female sex	2.015	0.324	12.517	0.452				
ECOG 2 or higher	2.368	0.241	23.304	0.460				
Hematologic disease risk: intermediate to high	20.294	1.968	209.315	0.011	14.537	1.720	122.881	0.014
Ileocecectomy or RHC	32.638	1.632	652.797	0.023	22.386	1.644	304.906	0.020
ANC < 1.00 (10^9^/L)	13.171	1.501	115.586	0.020	14.755	1.813	120.073	0.012
PLTs < 100 (10^9^/L)	11.371	1.763	73.355	0.011	10.080	1.872	54.277	0.007

POD, postoperative day; OR, odds ratio; LCI, lower confidence interval; HCI, higher confidence interval; ECOG, Eastern Cooperative Oncology Group; RHC, right hemicolectomy; ANC, absolute neutrophil count; PLT, platelet.

## Data Availability

Data is unavailable due to privacy and ethical restrictions.

## Supplementary Materials

The following supporting information can be downloaded at: https://www.mdpi.com/article/10.3390/cancers15205049/s1, Table S1: Hematologic Disease risk; Table S2: Clinical characteristics and surgical outcomes according to the hematologic disease risk; Table S3: Clinical characteristics according to the surgical outcomes; Table S4: Classification of complication.

## Abbreviations

CBC: complete blood count; RHC, right hemicolectomy; MAC, myeloablative conditioning; RIC, reduced intensity conditioning; ANC, absolute neutrophil count; Hb, hemoglobin; PLT, platelet; ECOG, Eastern Cooperative Oncology Group.
