# Supplementary material for: Risks Associated with Surgical Treatment for Appendicitis in Hematologic Patients
Source: Cancers (Basel). 2023 Oct 19;15(20):5049. doi: 10.3390/cancers15205049 (PMC10605880; doi:10.3390/cancers15205049)
Supplement: Supplementary file 1 [file cancers-15-05049-s001.zip › cancers-2622496-supplementary.pdf]

Supplemental Table S1. Hematologic Disease risk

Risk	Disease
Low	AML-favorable cytogenetics<br>CLL<br>CML
Intermediate	ALL<br>AML intermediate cytogenetics<br>MDS intermediate cytogenetics<br>MM
High	AML adverse cytogenetics<br>MDS adverse cytogenetics
Others	ITP

AML, acute myeloid leukemia; CLL, chronic lymphoblastic leukemia; CML, chronic myeloid leukemia; ALL, acute lymphoblastic leukemia; MDS, myelodysplastic syndrome; MM, multiple myeloma; ITP, immune thrombocytopenic purpura

Supplemental Table S2. Clinical characteristics and surgical outcomes according to the hematologic disease risk

Variables	Low (n = 31)	Intermediate (n = 35)	High (n = 7)	p-value
Hematologic disease				0.000
AML	11 (35.5)	15 (42.9)	2 (28.6)	
ALL	0 (0)	12 (34.3)	0 (0)	
MDS	2 (6.5)	0 (0)	0 (0)	
Lymphoma	3 (9.7)	1 (2.9)	5 (71.4)	
MM	0 (0)	7 (20)	0 (0)	
CML	13 (41.9)	0 (0)	0 (0)	
CLL	2 (6.5)	0 (0)	0 (0)	
Type of appendicitis				0.914
Simple	18 (58.1)	24 (68.6)	5 (71.4)	
Perforated	6 (19.4)	5 (14.3)	1 (14.3)	
Periappendiceal abscess	7 (22.6)	6 (17.1)	1 (14.3)	
Appendicolith	3 (9.7)	2 (5.7)	0 (0)	0.614
Type of surgery				0.389
Appendectomy	25 (80.6)	32 (91.4)	7 (100)	
Ileocecectomy	4 (12.9)	3 (8.6)	0 (0)	
Right hemicolectomy	2 (6.5)	0 (0)	0 (0)	
Approach				0.792
Open	2 (6.5)	2 (5.7)	0 (0)	
Laparoscopy	29 (93.5)	33 (94.3)	7 (100)	
Drain	18 (58.1)	17 (48.6)	3 (42.9)	0.652
OP time (min)	64 [50 – 103]	50 [40 – 80]	80 [70 – 85]	0.116
EBL (mL)	15 [10 – 30]	20 [5 – 50]	20 [5 – 50]	0.160
Initial ANC (10^9/L)				0.520
> 1.00	23 (74.2)	31 (88.6)	5 (71.4)	
0.50 ~ 1.00	4 (12.9)	1 (2.9)	1 (14.3)	
< 0.50	4 (12.9)	3 (8.6)	1 (14.3)	
Initial Hb (g/dL)				0.130
< 8.0	6 (19.4)	2 (5.7)	0 (0)	
Initial PLT (10^9/L)				0.276
> 100	20 (64.5)	27 (77.1)	4 (57.1)	
50 ~ 100	5 (16.1)	4 (11.4)	0 (0)	
< 50	6 (19.4)	4 (11.4)	3 (42.9)	
Preoperative ANC (10^9/L)				0.207
> 1.00	26 (83.9)	33 (94.3)	5 (71.4)	
0.50 ~ 1.00	1 (3.2)	0 (0)	1 (14.3)	

< 0.50	4 (12.9)	2 (5.7)	1 (14.3)	
Preoperative Hb (g/dL)				0.893
< 8.0	1 (3.2)	1 (2.9)	0 (0)	
Preoperative PLT (10^9/L)				0.777
> 100	20 (64.5)	27 (77.1)	5 (71.4)	
50 ~ 100	8 (25.8)	6 (17.1)	1 (14.3)	
< 50	3 (9.7)	2 (5.7)	1 (14.3)	
Total hospital stay (days)	6 [3 – 41]	5 [3 – 11]	9 [2 – 15]	0.759
Postoperative hospital stay (days)	5 [3 – 7]	5 [3 – 9]	3 [2 – 6]	0.489
Complication	3 (9.7)	4 (11.4)	2 (28.6)	0.380
In-hospital mortality	2 (6.5)	0 (0)	0 (0)	0.248

Data given as numbers (%) and median [interquartile range]

AML, acute myeloid leukemia; ALL, acute lymphoblastic leukemia; MDS, myelodysplastic syndrome; MM, multiple myeloma; CML, chronic myeloid leukemia; CLL, chronic lymphoblastic leukemia; OP, operation; EBL, estimated blood loss; ANC, absolute neutrophil count; Hb, hemoglobin; PLT, platelet.

Supplemental Table S3. Clinical characteristics according to the surgical outcomes

Variables	No complication (n = 65)	Complication (n = 10)	P-value
Age (year)	44 [28.5 – 60.5]	35 [28.5 – 50.0]	0.289
Sex			0.488
Male	38 (58.5)	7 (70)	
Female	27 (41.5)	3 (30)	
BMI (kg/m2)	22.0 [19.9 – 23.9]	22.6 [21.1 – 24.7]	0.370
ECOG			0.240
0 or 1	57 (87.7)	10 (100)	
2 or higher	8 (12.3)	0 (0)	
Hematologic disease			0.677
AML	23 (35.4)	5 (50)	
ALL	11 (16.9)	1 (10)	
MDS	2 (3.1)	0 (0)	
Lymphoma	8 (12.3)	1 (10)	
MM	7 (10.8)	0 (0)	
CML	11 (16.9)	2 (20)	
CLL	2 (3.1)	0 (0)	
ITP	1 (1.5)	1 (10)	

Data given as numbers (%) and median [interquartile range]

BMI, body mass index; ECOG, Eastern Cooperative Oncology Group; AML, acute myeloid leukemia; ALL, acute lymphoblastic leukemia; MDS, myelodysplastic syndrome; MM, multiple myeloma; CML, chronic myeloid leukemia; CLL, chronic lymphoblastic leukemia; ITP, immune thrombocytopenic purpura; OP, operation; EBL, estimated blood loss; ANC, absolute neutrophil count; Hb, hemoglobin; PLT, platelet.

Supplemental Table S4. Classification of complication

Clavien-Dindo Classification	Wound infection (n = 4)	Fluid collection / abscess (n = 2)	Ileus (n = 2)	Pneumonia (n = 1)	Fever (n = 1)
Grade I	2	1	2	0	0
Grade II	2	1	0	1	1
Grade III or IV	0	0	0	0	0

ANC, absolute neutrophil count; PLT, platelet.
